# Novel measure of lung function for assessing disease activity in asthma

**DOI:** 10.1136/bmjresp-2019-000531

**Published:** 2020-03-10

**Authors:** Nicholas M J Smith, John Couper, Christopher J Fullerton, Graham Richmond, Nick P Talbot, Gus Hancock, Ian Pavord, Grant A D Ritchie, Peter A Robbins, Nayia Petousi

**Affiliations:** 1 Department of Chemistry, Physical and Theoretical Chemistry Laboratory, University of Oxford, Oxford, UK; 2 Department of Physiology, Anatomy and Genetics, University of Oxford, Oxford, UK; 3 Oxford NIHR Respiratory Biomedical Research Centre, University of Oxford, Oxford, UK; 4 Nuffield Department of Clinical Medicine, Division of Experimental Medicine, Oxford, UK

**Keywords:** asthma

## Abstract

**Introduction:**

In asthma, lung function measures are often discordant with clinical features such as disease activity or control.

**Methods:**

We investigated a novel technique that provides a measure (σCL) of unevenness (inhomogeneity) in lung inflation/deflation. In particular, we compared σCL with FEV_1_% predicted (FEV_1_%pred) as measures of disease activity in the asthmatic lung.

**Results:**

σCL correlated modestly with FEV_1_%pred. However, σCL is not simply a proxy for FEV_1_%pred as the effects of salbutamol on the two parameters were unrelated. Importantly, σCL reflected disease control better than FEV_1_.

**Discussion:**

We conclude that σCL shows promise as an objective measure of disease activity in asthma.

**Key messages**

- In asthma, spirometric measurements associated with airways resistance are often discordant with other clinical features of disease activity or control.

- This study demonstrates that a novel technology measuring the evenness of lung expansion and contraction can reflect these clinical features better than spirometry.

- A reliable, objective measure of disease activity in asthma would be very valuable for both patient management and clinical research.

## Introduction

In asthma, lung function measures, such as spirometry, are often discordant with the clinical assessment of disease activity, as determined by symptoms, exacerbation frequency and response to treatment.^1 2^ There is no single diagnostic test for asthma, and both clinical assessment of symptoms and objective tests can produce false positives and false negatives.^3^ Spirometry may be normal in patients with active airways disease, and the diagnosis of asthma, for example, may require multiple measurements over time to demonstrate variable airflow obstruction. In addition, age-related changes in FEV_1_ or fixed airflow obstruction may lead to overdiagnosis or treatment in older people. This disparity, alongside the fact that primary care clinicians may not have access to reliable lung function testing at the point of clinical decision-making, often leads clinicians to adopt a no-test approach to diagnosis and treatment.

Recently, Mountain *et al.* described a new approach to lung function testing that involved assessing the inhomogeneity of gas exchange in the lung.^4^ This study is a first look at whether this technique has the potential to provide a better measure of disease activity in the lungs of asthmatic patients than standard spirometry.

## Methods

### Patient and public involvement

Research into novel diagnostics for asthma is one of the research priorities for a leading UK asthma patient group. Our experimental protocol was designed after obtaining feedback from patients during pilot studies in order to optimise tolerability and acceptability during testing and in future clinical practice. Informal feedback was obtained from all participants in the current study to inform future experimental design.

### Experimental methods

Seventeen patients with asthma, recruited from a hospital-based asthma clinic, and 17 healthy volunteers were studied (see online supplementary file for details). Each patient underwent standard forced spirometry and a lung inhomogeneity test before and 30 min after bronchodilation with inhaled salbutamol (400 μg via a spacer).

10.1136/bmjresp-2019-000531.supp1 Supplementary data

The lung inhomogeneity tests were performed using molecular flow sensing technology^5^ that uses laser absorption spectroscopy and provides highly precise molar flows for oxygen, carbon dioxide and nitrogen at the mouth. Participants breathed air for 10 min and then pure oxygen for 5 min through a mouthpiece connected to the molecular flow sensing device.

**Figure 1:**
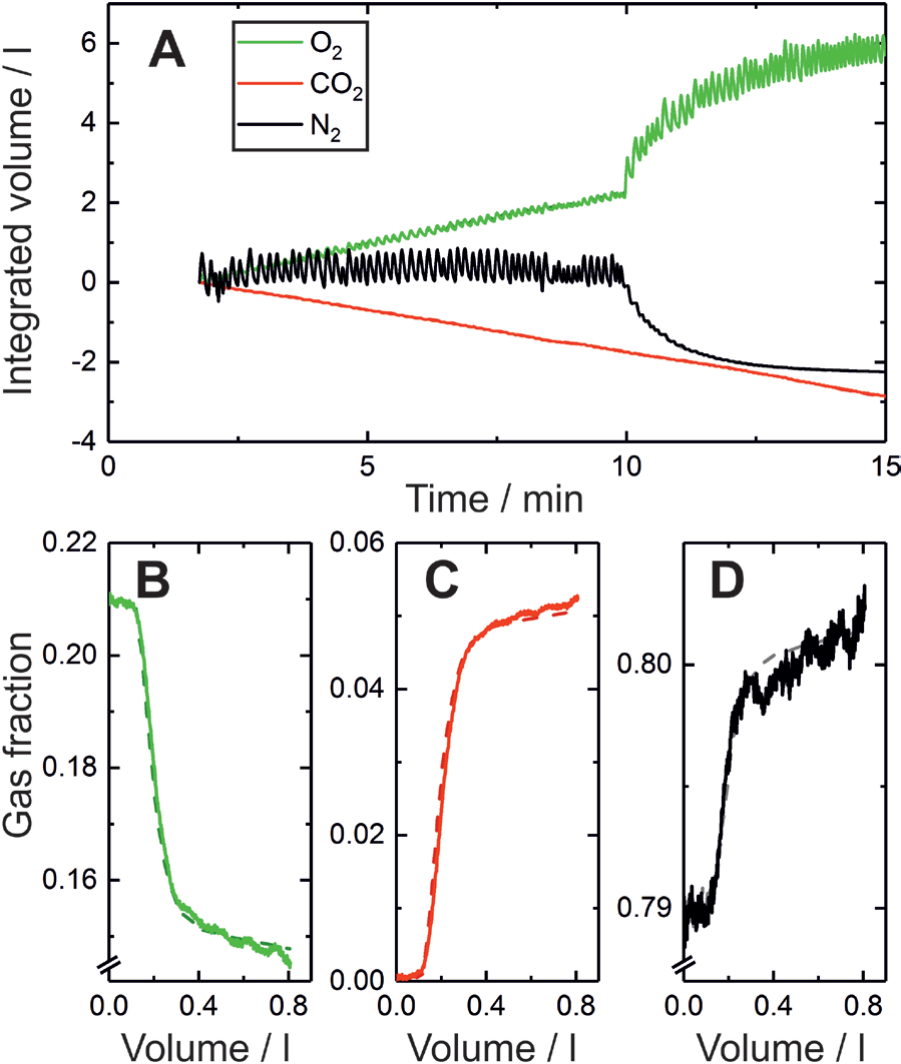
Example recording of lung inhomogeneity measurement and model fit. (A) Tidal gas flows at the mouth for nitrogen (black), oxygen (green) and carbon dioxide (red) over a 10 min period of breathing air, followed by a 5 min period of breathing pure oxygen, as recorded using the in-airway molecular flow sensor every 10 ms.^4^ Also plotted (broken lines) for each gas are the fits of the model to the data, but these are obscured because the quality of fit is so high. (B)–(D) measured expirogram records for a single representative breath during the air-breathing phase for (B) oxygen, (C) carbon dioxide and (D) nitrogen gas fractions. The broken lines indicate the gas fractions calculated by the model.

A computational model of an inhomogeneous lung was fit to the gas-exchange data^4^ (figure 1). Briefly, the model is comprised of a ‘lung’ with 125 lung units, each with an equal share of the total volume at functional residual capacity (FRC), but differing in their fractional share of total lung compliance, of total pulmonary vascular conductance and of total deadspace. The process of fitting the model to the data is based on the principle of mass balance and provides estimates of anatomical deadspace, alveolar volume at FRC and three measures of inhomogeneity. Two of the inhomogeneity measures are σCL and σCd, which are the SDs for the log-normal distributions of (standardised) alveolar compliance and vascular conductance across the lung volume, respectively. The third measure is σVD, which is the SD for the normal distribution for the (standardised) deadspace across the lung volume. Further details are given in the study by Mountain *et al*.^4^ The present study focuses particularly on σCL as a measure of unevenness of lung inflation/deflation during breathing.

### Data analysis

The following analyses were conducted on the data: (1) values for σCL and other model parameters were compared between the healthy volunteers and asthma patients; (2) the correlation between σCL and FEV_1_% predicted (FEV_1_%pred) was calculated; (3) the effects of salbutamol on σCL and FEV_1_%pred were compared; (4) the relationship between symptom severity (as assessed by the patients’ clinicians using the ACQ5 asthma control questionnaire) and σCL was explored and (5) the ability of σCL versus FEV_1_%pred to predict overall disease control was examined. A pragmatic approach was used to define disease control based on whether the clinician intended to escalate therapy (‘bad control’) or not (‘good control’), based on their overall assessment.

Pearson correlation coefficients were used to explore relationships/correlation between variables. A Shapiro–Wilk test of normality was performed on the data and Student’s unpaired t-tests were used to compare parameter values between healthy versus asthma groups. Logistic regression analysis was used to explore the predictive power of σCL versus FEV_1_%pred in terms of disease control.

## Results

**Figure 2:**
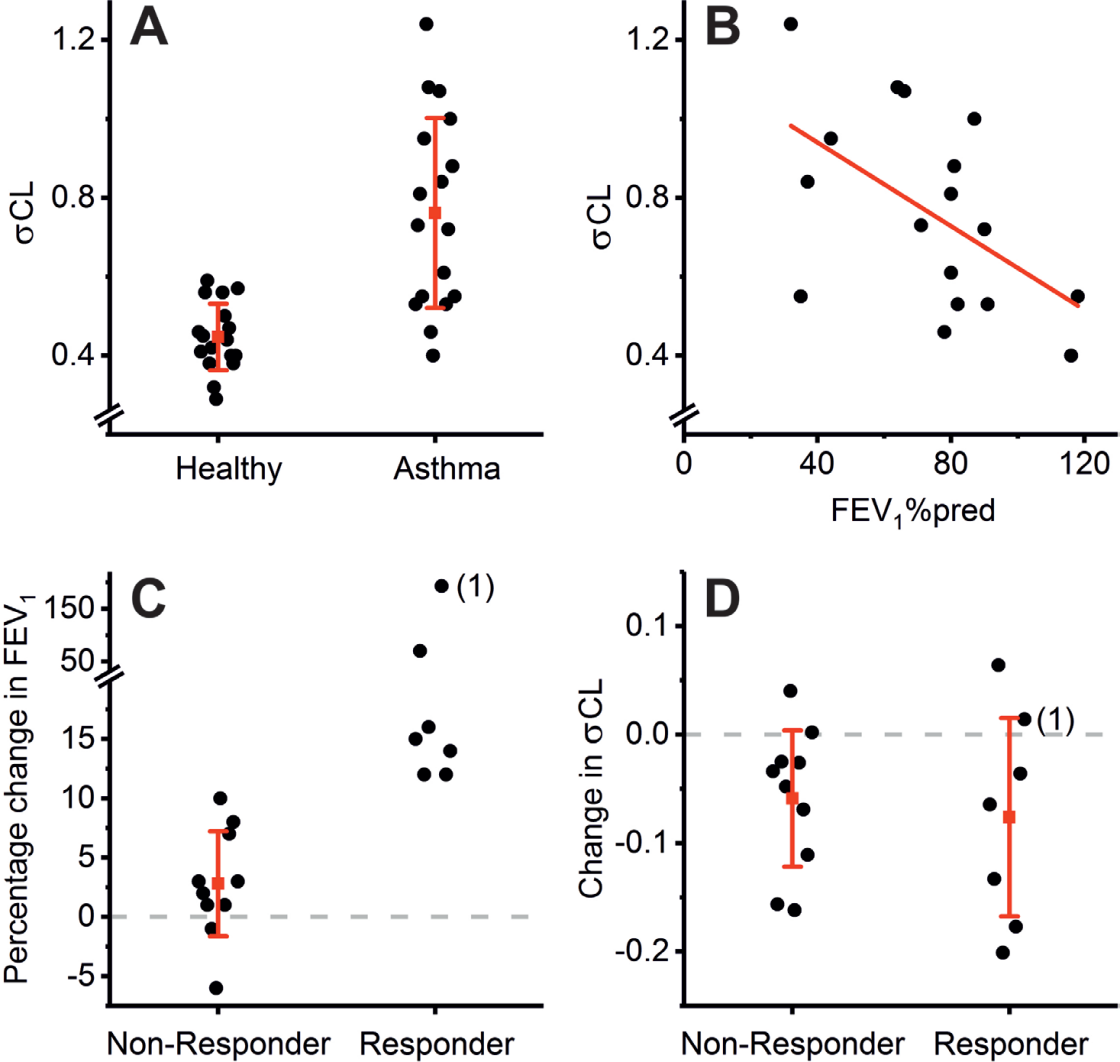
σCL is not a proxy measurement for FEV_1_% predicted (FEV_1_%pred). (A) σCL values for healthy controls and patients with asthma. The average value for σCL is higher in the asthma group than in the control group (0.762±0.241 vs 0.447±0.084, respectively, mean±SD, p<0.001 Student’s t-test). (B) Relationship between σCL and FEV_1_%pred for the asthma group. The correlation is significant (Pearson’s r=−0.54, p<0.05) but it leaves 71% of the variance in σCL unexplained. (C)Effect of bronchodilation with salbutamol on FEV_1_ in asthma. The asthma patients have been divided into ‘responders’ and ‘non-responders’ based on their degree of bronchodilator reversibility (responders exhibit an effect size greater than 12% with a minimum increase in FEV_1_ of 200 mL). The patient labelled (1) is discussed in the Results. (D) Effect of bronchodilation with salbutamol on σCL in asthma. The asthma patients have again been divided on the basis of their FEV_1_ response, as described in (C). Note that salbutamol reduces σCL in both groups, but the effects do not differ between ‘responders’ and ‘non-responders’. In keeping with this finding, there was an absence of significant correlation (r=0.10, p=0.70) between the effect size of salbutamol on FEV_1_ and the effect size on σCL. For (A), (C) and (D), red symbols and lines represent means and SD, respectively. For (A) and (B), data are pre-salbutamol; post-salbutamol data are similar and are given in the online supplementary file.

Values for σCL were significantly larger in asthma patients compared with healthy volunteers (figure 2A). Values for other model parameters and FEV_1_%pred are provided in the online supplementary file.

There was a significant correlation between σCL and FEV_1_%pred (figure 2B), but the majority of the variance (71%) in σCL was unexplained by FEV_1_%pred. Following salbutamol, σCL fell in the patients with asthma, but the effects of salbutamol on σCL were independent of whether the patients showed FEV_1_ bronchodilator reversibility (figure 2C, D). Indeed, for patient 1 in figure 2, FEV_1_ rose following salbutamol by 193% while σCL hardly changed (0.55 to 0.57), demonstrating that σCL is not a surrogate for FEV_1_.

**Figure 3:**
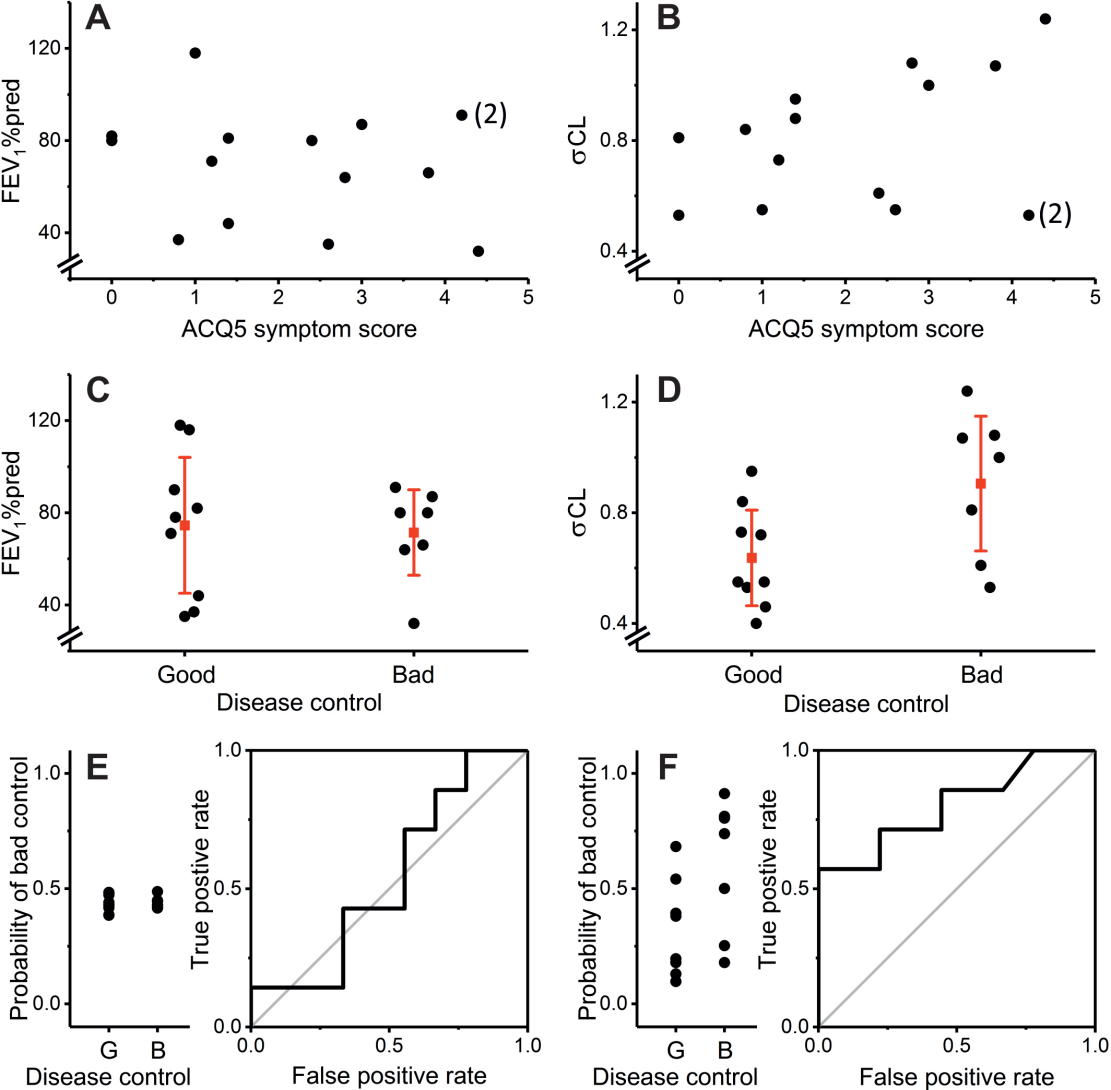
σCL reflects disease activity more tightly than FEV_1_% predicted (FEV_1_%pred). (A) and (B) FEV_1_%pred and σCL as a function of ACQ5 asthma control questionnaire score, respectively. Neither variable correlated significantly with symptoms (Pearson’s r=−0.27, p=0.40 and r=0.43, p=0.15 for FEV_1_%pred and σCL, respectively). The patient labelled (2) is considered further in the Discussion. (C) and (D) FEV_1_%pred and σCL by physicians’ assessment of ‘disease control’, respectively. ‘Good control’ was defined as therapy either unchanged or reduced at clinic visit, ‘bad control’ was defined as therapy increased at clinic visit. There was no significant difference in FEV_1_%pred between the two groups (p=0.81 Student’s t-test). σCL was significantly higher in the ‘bad control’ group compared with the ‘good control’ group (p<0.05). Red symbols and lines represent means and SD, respectively. (E) and (F)Logistic regressions to predict ‘disease control’ using FEV_1_%pred or σCL as predictors, respectively. σCL was the better predictor, as judged by the probabilities for individual patients (left panels) and area under the curve of the receiver–operator plots, which were 0.540 for FEV_1_%pred and 0.802 for σCL. Data illustrated are pre-salbutamol. Post-salbutamol data are similar and are given in the online supplementary file.

Figure 3A, B illustrates that neither FEV_1_ nor σCL correlated significantly with symptom severity in the patients. For an index of ‘disease control’, we defined control as ‘bad’ if the physician at the clinic visit before measurement deemed that an increase/escalation in therapy was necessary (either increased therapy on the day or referred further for biologic therapy). Control was defined as ‘good’ in all other patients, where the physician felt no therapy escalation was needed and either decreased or left unchanged a patient’s therapy. There was no significant difference in FEV_1_%pred between patients with ‘good control’ versus those with ‘bad control’ (figure 3C). In contrast, σCL was significantly higher in patients with ‘bad control’ than those with ‘good control’ (figure 3D). Consistent with this, ACQ5 score was 1.17±0.85 (mean±SD) in the ‘good control’ group and 2.94±1.50 in the ‘bad control’ group (p<0.05). Figure 3E, F illustrates an analysis using logistic regression which demonstrates that σCL is a better predictor of disease control than FEV_1_%pred.

## Discussion

The results indicate that σCL is not simply a proxy for FEV_1_%pred, but rather that it captures different aspects of the disease’s pathophysiology, beyond airflow obstruction. FEV_1_ changes are generally thought to arise from hyper-reactivity of smooth muscle in the large airways resulting in increased airways resistance. In contrast, σCL may preferentially reflect the effects of hyper-reactivity of smooth muscle in the small airways through an effect on ventilation distribution. An alternative hypothesis is that σCL reflects small airways inflammation. Small airways inflammation is associated with localised oedema which increases the stiffness of that part of the lung. As the distribution of disease across the lung tends to be uneven, then so too is the distribution of stiffness. This mechanism can explain an increase in σCL without invoking any change in airways resistance. Indeed, distinct mechanisms of action of salbutamol on σCL (enhanced lung water clearance) and FEV_1_ (smooth muscle relaxation in large airways) may explain why the effects of salbutamol on σCL were similar for both FEV_1_ responders and non-responders to salbutamol.

Neither FEV_1_%pred nor σCL correlated significantly with symptoms. Patient 2 (figure 3) had a very high symptom score but had normal values for σCL and FEV_1_%pred (0.53 and 91%, respectively). On review of these patients’ clinical records, their symptoms appeared to have a multifactorial origin including significant nasal/upper airway symptoms, breathlessness from hyperventilation/dysfunctional breathing, depression and fibromyalgia. This patient demonstrates the value that an objective measure of disease activity within the lung could have in managing asthma. Apart from this patient, the four patients with the highest symptom scores also had the highest σCL values. Indeed, without this outlier, the correlation between σCL and symptoms would have been significant (p<0.02 and p<0.01, pre-salbutamol and post-salbutamol, respectively). Unlike FEV_1_, σCL predicted whether the physician deemed an escalation of therapy necessary. This is consistent with the hypothesis that σCL reflects small airways disease, which is increasingly recognised as associated with severe refractory asthma.^6 7^

The technique used in this study was developed to quantify physiological aspects of lung function that cannot be obtained through standard lung function testing. To achieve this, the novel measurement technology^4^ was used to provide continuous, highly precise measurements of molar gas flows at the mouth. This precision, combined with the principles of mass balance, enables measurements of gas flow at the mouth to be linked via a computational model to the underlying physical properties of the lung, including the distribution of compliance.

Other physiological techniques that assess small airway function, including oscillometry and single or multiple exhaled breath analyses, have been used in the research setting for some years, and more recently evaluated specifically in asthma,^8^ but none has yet been adopted into routine clinical practice. This may reflect the considerable variability that has been associated with these alternative measures.^9 10^ The novel highly accurate gas analysis underlying our approach improves accuracy and reproducibility and allows the provision of indices that directly relate to underlying physiological properties of the lung. Although technically sophisticated, the test is simple to undertake for both the operator and the patient, with no forced breathing manoeuvres required. It is non-invasive, does not involve ionising radiation and does not require expensive equipment and reagents such as MRI scanners and scarce isotopes. Consequently, it is well suited to clinical use. While the current study involved only a small number of participants and is preliminary, the results are promising and suggest that the method may provide a powerful new objective measure of disease activity in the lung. To determine whether this early promise is fulfilled, and if so, whether the measurement is useful in the management of asthma, will require larger studies across different patient populations using longitudinal and interventional designs and further comparisons with other available lung function techniques.
