# Supplementary material for: Novel measure of lung function for assessing disease activity in asthma
Source: BMJ Open Respir Res. 2020 Mar 10;7(1):e000531. doi: 10.1136/bmjresp-2019-000531 (PMC7066624; doi:10.1136/bmjresp-2019-000531)
Supplement: Supplementary data [file bmjresp-2019-000531supp001.pdf]

Supplementary Material

A novel measure of lung function for assessing disease severity in asthma

Nicholas M J Smith, John Couper, Christopher J Fullerton, Graham Richmond,
Nicholas P Talbot, Gus Hancock, Ian D Pavord, Grant A D Ritchie,
Peter A Robbins, Nayia Petousi

Smith NMJ, *et al. BMJ Open Resp Res* 2020; 7:e000531. doi: 10.1136/bmjresp-2019-000531

Table of Contents

Table of Contents **2**

1. Supplementary Text **3**

2. Figures **4**

Figure S1. FEV_1 % predicted (FEV_1%pred) and model parameters, other than σCL, for healthy participants and for patients with asthma. **4**

Figure S2. σCL is not a proxy measurement for FEV_1%pred following bronchodilation. **5**

Figure S3. σCL reflects disease activity more tightly than FEV_1%pred following bronchodilation. **6**

Smith NMJ, *et al. BMJ Open Resp Res* 2020; 7:e000531. doi: 10.1136/bmjresp-2019-000531

1. Supplementary Text

17 patients with asthma and 17 healthy participants were studied, with age of 57.8 ± 13.9 years (mean ± SD) and 50.6 ± 17.3 years, respectively; height of 1.68 ± 0.08 m and 1.73 ± 0.10 m, respectively; and, weight of 82 ± 19 kg and 70 ± 16 kg, respectively. Eight out of 17 of the asthma patients were female (47%) while 6 out of the 17 participants were female (35%) in the healthy group. Fourteen out of 17 of the asthma patients were on Step 4 or 5 asthma treatments[8].

For the salbutamol reversibility test, patients were asked to refrain from inhaled maintenance therapies for at least 12 hours (24 hours for once-daily preparations) and short-acting bronchodilators at least 4 hours before attending the hospital.

Shapiro-Wilk tests were first performed on the data to check that there were no significant deviations from normality (for FEV_1%pred, $p>0.2$ and $p>0.96$, and for σCL, $p>0.49$ and $p>0.56$, in the asthma and control groups, respectively). Student's unpaired t-tests were then used to compare parameter values between healthy versus asthma groups.

Figure S1 shows values for FEV_1 % predicted (FEV_1%pred) together with model parameters other than σCL for healthy participants and patients with asthma. Figure S2 shows post-bronchodilation values for σCL and FEV_1%pred. Figure S3 shows the analysis of the relationship of post-bronchodilation values of σCL and FEV_1%pred with symptoms and "disease control".

Smith NMJ, *et al. BMJ Open Resp Res* 2020; 7:e000531. doi: 10.1136/bmjresp-2019-000531

2. Figures

Figure S1. FEV_1 % predicted (FEV_1%pred) and model parameters, other than σCL, for healthy participants and for patients with asthma.

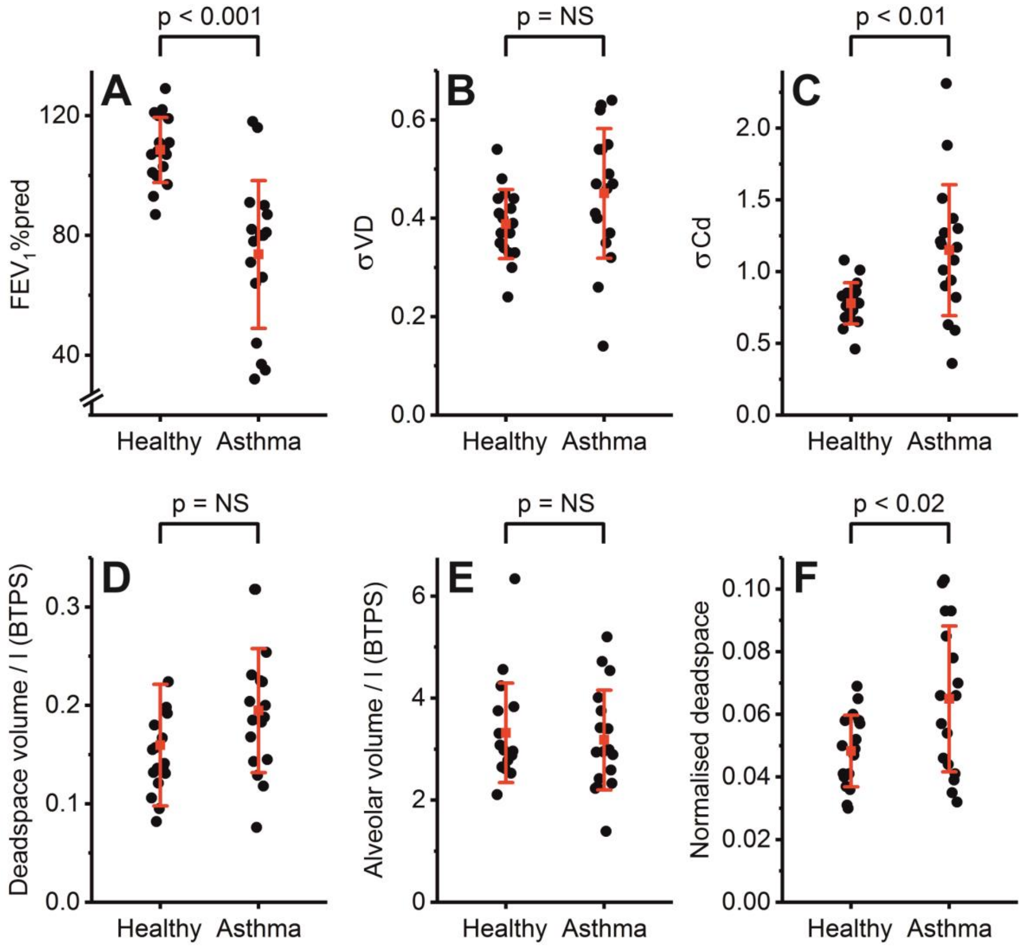

(A) FEV_1%pred. **(B)** Standard deviation for the distribution of standardised anatomical deadspace across the lung volume, σVD. **(C)** Standard deviation for the distribution of standardised vascular conductance across the lung volume, σCd. **(D)** Anatomical deadspace volume for healthy controls and patients with asthma. **(E)** Alveolar volume at functional residual capacity. **(F)** Anatomical deadspace normalised to alveolar gas volume at functional residual capacity. Significant differences were detected (Student's t-tests) between the asthma patients and healthy controls for FEV_1%pred, σCd and normalised anatomical deadspace. Red symbols and lines represent means and SD, respectively.

Smith NMJ, *et al. BMJ Open Resp Res* 2020; 7:e000531. doi: 10.1136/bmjresp-2019-000531

Figure S2. σCL is not a proxy measurement for FEV_1%pred following bronchodilation.

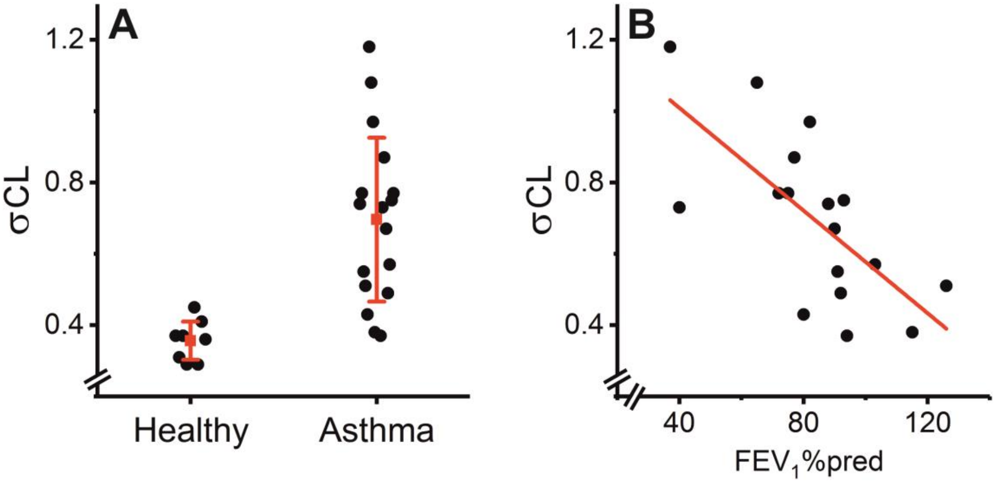

(A) σCL values for healthy controls and patients with asthma. Red symbols and lines represent means and SD, respectively. The average value for σCL is higher in the asthma group than in the control group (0.696±0.230 versus 0.356±0.054, mean±SD, respectively, p<0.001 Student's t-test). Note, 8 of the 17 healthy participants also undertook the post-salbutamol study. **(B)** Relationship between σCL and FEV_1%pred for the asthma group. The correlation is significant (Pearson's r=-0.69, p<0.005), but leaves 52% of the variance in σCL unexplained.

Smith NMJ, *et al. BMJ Open Resp Res* 2020; 7:e000531. doi: 10.1136/bmjresp-2019-000531

Figure S3. σCL reflects disease activity more tightly than FEV_1%pred following bronchodilation.

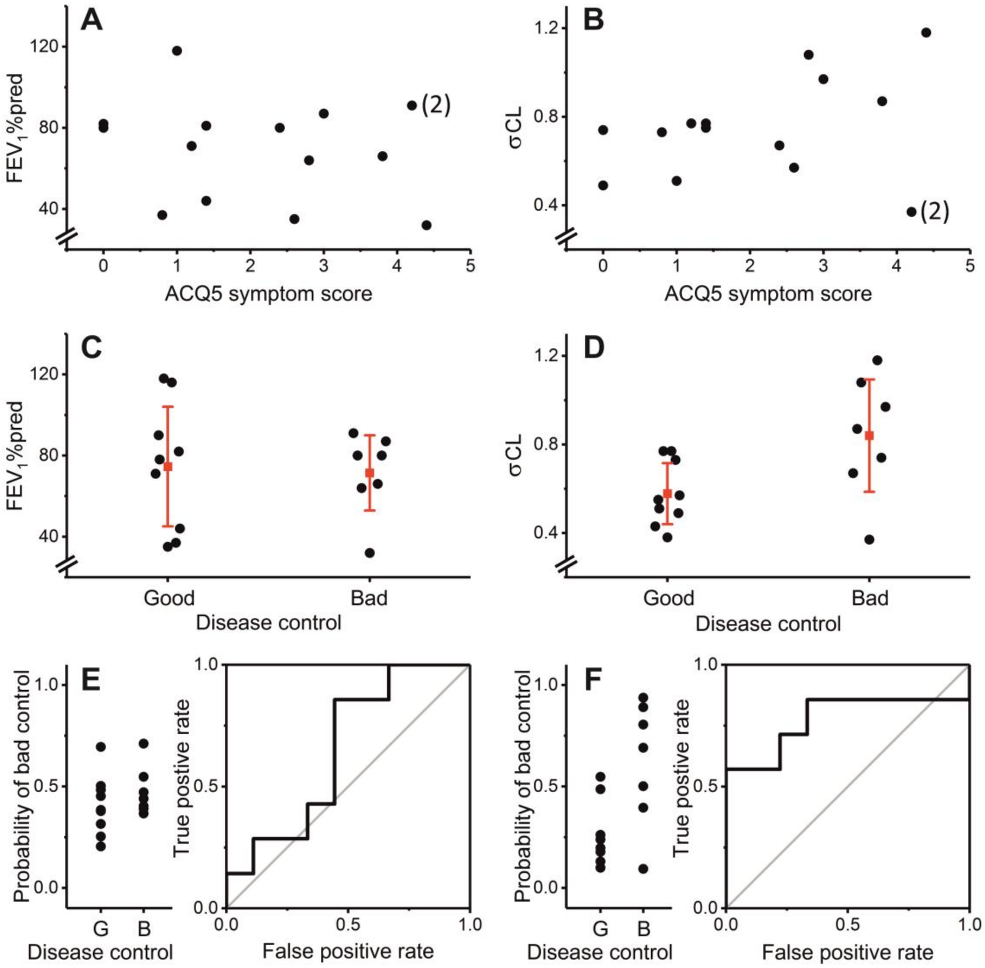

(A),**(B)** FEV_1%pred and σCL as a function of asthma symptom questionnaire (ACQ5) score, respectively. Neither variable correlated significantly with symptoms (Pearson's r=-0.24, p=0.40 and r=0.36, p=0.21, for FEV_1%pred and σCL, respectively). The patient labelled (2) is considered further in the Discussion. **(C)**,**(D)** FEV_1%pred and σCL by "disease control", respectively. "Good control" was defined as therapy either unchanged or reduced at clinic visit, "bad control" was defined as therapy increased at clinic visit. There was no significant difference in FEV_1%pred between the two groups (p=0.30 Student's t-test). σCL was significantly higher in the "bad control" group compared with the "good control" group ($p<0.05$). Red symbols and lines represent means and SD, respectively. **(E)**,**(F)** Logistic regressions to predict asthma control using FEV_1%pred or σCL as predictors, respectively. σCL was the better predictor, as judged by the probabilities for individual patients (left panels) and area under the curve of the receiver-operator plots, which were 0.65 for FEV_1%pred and 0.78 for σCL.

Smith NMJ, *et al. BMJ Open Resp Res* 2020; 7:e000531. doi: 10.1136/bmjresp-2019-000531
